# External insect gall morphology influences the functional guilds of natural enemy communities

**DOI:** 10.1098/rspb.2024.2424

**Published:** 2024-12-11

**Authors:** Quinlyn Baine, Daniel W. W. Hughes, Emily E. Casares, Ellen O. Martinson, Vincent G. Martinson

**Affiliations:** ^1^ Department of Biology, University of New Mexico, Albuquerque, NM 87131, USA

**Keywords:** parasitoid, hypothesis, galler, inducer, koinobiont, idiobiont

## Abstract

The evolution of diverse and novel morphological traits is poorly understood, especially how symbiotic interactions can drive these adaptations. The extreme diversity of external traits in insect-induced galls is currently explained by the Enemy Hypothesis, in which these traits have selective advantage in deterring parasitism. While previous tests of this hypothesis used only taxonomic identity, we argue that ecologically functional traits of enemies (i.e. mode of parasitism, larval development strategy) are a crucial addition. Here, we characterize parasitoid guild composition across four disparate gall systems and find consistent patterns of association between enemy guild and gall morphology. Specifically, galls with a longer average larva-to-surface distance host a significantly higher proportion of enemies with a distinct combination of functional traits (i.e. ectoparasitic, idiobiont, elongate ovipositor). Our results support the Enemy Hypothesis and highlight the importance of species ecology in examining insect communities and the evolution of novel defensive characters.

## 1. Introduction

Symbiotic interactions are responsible for many major innovations, including shaping the evolution of entire ecological guilds (e.g. mycorrhizae, pathogens), allowing lineages to expand their niche space to exploit novel resources (e.g. endosymbiotic microbes of specialist herbivorous insects) and facilitating the expansion of species to new ecosystems (e.g. plants and terrestrial fungi) [1–5]. However, an often-overlooked aspect of symbiotic interactions is their potential to create unique habitats that can then be exploited by other organisms (e.g. corals). While the term ‘ecosystem engineer’ is traditionally applied to a single species, genotype-by-genotype interactions between symbiotic partners that modify, create or maintain habitats can dramatically increase overall ecosystem biodiversity [6]. However, other organisms that utilize niches generated by ecosystem engineer symbionts, such as predators, can also apply selective pressures on the interacting hosts [7]. Among the most critically understudied examples of symbiotic ecosystem engineers are insects that induce plant galls—structures that provide nutrition and protection for the inducer’s offspring, but also serve as habitat for highly specialized communities of associated parasitoids, hyperparasitoids, predators and inquilines [8–11].

**Figure 1.**
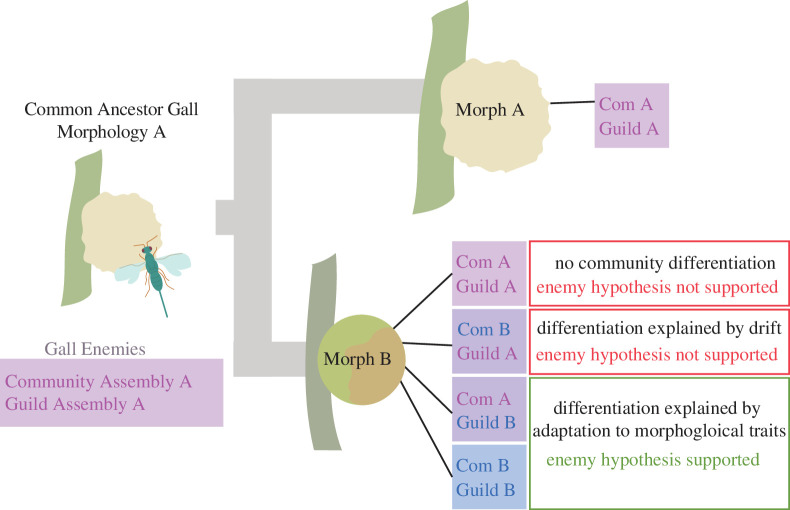
For the Enemy Hypothesis to be supported, there must be an observable shift in communities from one gall morphology to another in functional guild assembly, not just taxonomic assembly, as this suggests adaptations inhibit ancestral enemies, but may create a niche for a new enemy assemblage.

Though a gall is made of plant tissue, its morphology is directed by the inducer [10,12]. Galls display unique structures, colours, gene expression patterns and chemicals not observed elsewhere in the plant, making them complex and novel organs [13]. Whereas the internal gall tissue, which provides nutrition for the developing offspring, is generally consistent across many galling taxa [9,14], external gall traits are highly diverse even among closely related gall inducers [10,15]. Exterior traits, such as hairs and chemical exudates, have been hypothesized to represent adaptations for predator deterrence [14,16,17] and several studies have found that these traits can predict enemy community structure [10] and rate of attack [15]. Formalized as the ‘Enemy Hypothesis’, this idea predicts that modifications in gall structure function primarily to defend against predatory organisms that target galls—chiefly parasitoid wasps that lay their eggs on or in the primary gall inducer, eventually causing its death [9,10,16,17]. This hypothesis is supported by studies that find (i) higher survival rates of the gall inducer are correlated with variation in gall traits within species and certain convergent traits among species (e.g. hairiness, external tissue hardness, size) [9,10,15,17–20] and (ii) a relationship between a parasitoid’s ovipositor length and attacked gall size [21,22]. However, previous analyses have only directly tested the Enemy Hypothesis by documenting changes in parasitoid presence/absence, and taxonomic composition of the enemy community [10,19,23–25], which could be explained by different community assembly processes (figure 1). These tests neglect important aspects of parasitoid ecology (i.e. mode of parasitism, larval development strategy, phenology of attack) [26–28] and assume that all enemy species have functionally equivalent attacks on the host gall.

Parasitoids are an extremely species-rich group, and consequently employ various strategies to access and kill hosts [28]. Species with similar strategies form a functional guild that can be shared by members of disparate taxonomic groups [26,27,29]. In gall parasitoids, these guilds define the interaction with the extended phenotype (plant gall) of the inducer [8]. Certain external traits of a gall are better at deterring particular enemy guilds. For example, thick-walled galls will defend well against parasitoids with short ovipositors, but poorly against parasitoids with long ovipositors [7]. However, phenological adaptations of the parasitoid attack can overcome some defences. For example, short ovipositor parasitoids can be successful during early gall development before the thick gall walls are fully formed. The Enemy Hypothesis predicts that the evolution of novel external gall traits should decrease susceptibility to parasitism; therefore, we clarify the Hypothesis to state that a gall morphology will become adaptive if it protects against members of a previously problematic enemy guild. However, the exclusion of one guild may leave a gall susceptible to colonization by members of another guild. Therefore, we propose that, to support the Enemy Hypothesis, a change in gall morphology must result in a corresponding change in the ecologically functional traits of the enemy community (illustrated in figure 1).

To directly compare external gall morphology and enemy communities, we determine the taxonomy and functional ecology of the natural enemies associated with *different* gall morphs in: (i) two sympatric sister species in the genus *Aciurina* (Tephritidae), (ii) a closely related pair of rose gall wasps in the genus *Diplolepis* (Diplolepididae), (iii) alternate generations of a single oak gall wasp species (Cynipidae) and (iv) a comprehensive continental-scale sampling of willow-galling sawflies (Tenthredinidae); and across *similar* gall morphs in the ‘leaf pea’ willow-galling sawflies.

This approach not only tests the validity of the Enemy Hypothesis but also elucidates how gall traits may deter different functional guilds of enemies, providing insight into the complex ecological interactions that drive the evolution of novel traits in galling insects. Moreover, understanding these dynamics has broader implications for ecological and evolutionary theory, as well as for biodiversity conservation, as it highlights the importance of species interactions in shaping ecosystems. We aim to fill a critical gap in our knowledge by systematically testing the Enemy Hypothesis and to clarify the existing Enemy Hypothesis of gall evolution with the addition of parasitoid functional guild as a consideration that must be made in community evaluation.

## 2. Methods

### (a) Community composition

**Figure 2.**
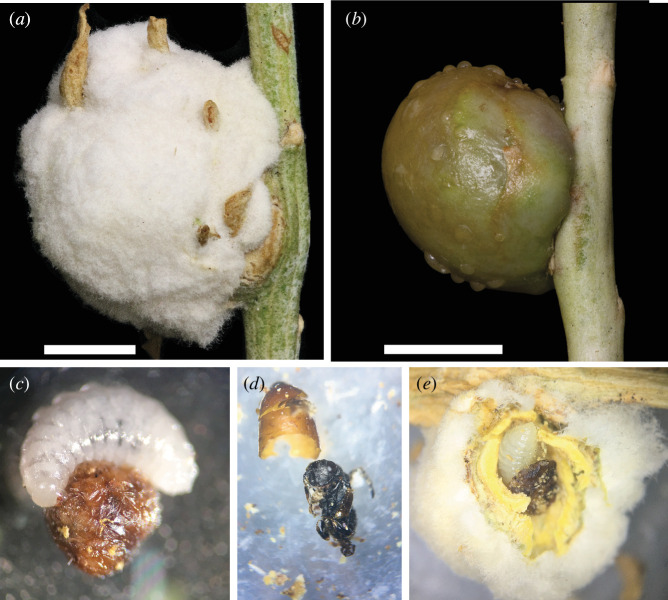
(*a*) *Aciurina bigeloviae* gall, and (*b*) *Aciurina trixa* gall. White scale bars = 5 mm. Parasitoid immatures and modes of parasitism and development. (*c*) *Eurytoma bigeloviae* larva feeding on an *A. bigeloviae* larva. (*d*) *Eurytoma chrysothamni* pupa removed from the interior of the *A. trixa* exuvium above. (*e*) *Torymus capillaceus albitarsis* larva feeding on *A. bigeloviae* larva inside gall central chamber. Photo credit Q. Baine.

To test this new prediction of the Enemy Hypothesis and demonstrate the methods used to characterize enemy guilds in a novel galling system, we first examined the associated communities of sister gall-inducing fly species *Aciurina bigeloviae* (Cockerell 1890) and *Aciurina trixa* Curran 1932 (Diptera, Tephritidae). These two species are mostly allopatric in New Mexico but are sympatric in the northern Rio Grande Valley. Both *A. bigeloviae* and *A. trixa* are univoltine and generate single-chambered spherical galls on *Ericameria nauseosa* (Pall. ex Pursh) G.L. Nesom & G.I. Baird (Asteraceae). These two species are distinct from one another in two notable ways: (i) host plant variety specificity and (ii) gall external morphology. In New Mexico, *A. bigeloviae* induces a densely tomentous gall, similar in appearance to a cotton ball, on *E. nauseosa* ssp. *nauseosa* var. *graveolens*, and *A. trixa* induces a smooth and resinous gall on *E. nauseosa* ssp. *nauseosa* var. *latisquamea* (figure 2*a,b*). The gall morph difference between these two species is striking and may be highly influential in defining the gall-associated arthropod community composition. The thick layer of cotton-like fibres on *A. bigeloviae* galls increases the distance from the gall surface to the inner larval chamber, whereas the resin on *A. trixa* galls is frequently sticky and/or waxy. Both of these traits have the potential to limit successful parasitoid oviposition, but they do so through different mechanisms and may be protective—and correspondingly susceptible—to different parasitoid guilds.

For the characterization of the full aggregate community of *A. bigeloviae* and *A. trixa*, we followed the methods published in [30]. Briefly summarized, we collected 200 galls from each of seven New Mexico populations of each species in the summer of 2021 and 2022 (3800 galls total), and reared all in the laboratory within individual vials. Each emerged insect was recorded, counted and identified to genus or species using morphology and mitochondrial *cytochrome oxidase subunit b* barcode sequencing of representative specimens. Life history was determined by collecting a selection of galls haphazardly from the same sites earlier in the season and dissecting them to observe larval interactions, then sequencing a subset of larvae to confirm identity. Representative barcodes are deposited in GenBank (OR336222−34, OR438293−95).

To perform community composition analyses, we aggregated species counts per gall into plant groups (20 galls per plant and 10 plants per site). To test gall morph as a predictor of community composition with site and individual plant nested in site, as random effects, we used a permutational multivariate ANOVA (PERMANOVA) with Bray−Curtis distances and 9999 permutations using PRIMER-E [31] software with the PERMANOVA+ add on [32] to enable a nested model design. The following analyses were performed using R v. 4.1.2 [33]. For ordination of communities, we used two-dimensional non-metric multi-dimensional scaling (NMDS) using Bray−Curtis distances and 500 maximum random starts and report stress values (*metaMDS*, package ‘vegan’ [34]) and present fit of model on ordination estimated with 9999 permutations (*envfit*, package ‘vegan’). To test gall morph as a predictor of abundance and richness, we aggregated species counts into sites per year (*n* = 20) and used generalized mixed effects models (*glmer.nb*; package ‘lme4’ [35]) with a negative binomial distribution to control for overdispersion and site as a random effect. Model fit was confirmed by normality of residuals and homogeneity of variances (package ‘DHARMa’). Where assumptions for overdispersion were not met, we used non-parametric Kruskal−Wallis rank sum tests (*kruskal.test* [33]). Dissimilarity indices with Bray-Curtis distances were calculated and used in multivariate homogeneity of groups dispersions analysis to find measures of beta-diversity by gall morph (*vegdist, betadisper*; package ‘vegan’) as represented by distance-from-centroid and compared using ANOVA.

### (b) Parasitoid guild characterization

To categorize the guild (as defined in [27]) for each parasitoid species identified in this study, we selected three factors: (i) mode of parasitism: an *endoparasitoid* deposits eggs inside the host body cavity and an *ectoparasitoid* deposits on the surface or outside the host body, (ii) larval development strategy: a *koinobiont* parasitoid keeps its host alive to feed on an actively developing host, and an *idiobiont* halts development to feed on a dead or paralysed host, and (iii) adult female ovipositor length. We largely followed Mills' [26] guild definitions for parasitoids of holometabolous insects, but used adult ovipositor length as a simplified proxy for host stage (i.e. phenology of attack)—an elongate ovipositor enables access to a mature host inside a gall (late host stage) and a short ovipositor requires an alternative path of access (e.g. attacking an egg or early instar host before gall structure reaches ultimate form) (early host stage).

We identified the mode and strategy by performing dissections of haphazardly collected galls throughout the year and observing evidence of larval behaviour. For example, we were able to identify a koinobiont if the host had developed a pupal skin prior to emergence of the adult parasitoid (figure 2*d*). Where species were too uncommon to be found in haphazard collection, we characterized these factors from knowledge of the taxon in the literature.

To determine mean ovipositor length, three to six females of each of the six most common parasitoid species from each gall morphology, where present in sufficient abundance, were dissected (*n* = 43). We removed each whole gaster and soaked it in 5% w/v potassium hydroxide solution for 1 h at 50°C, then rinsed it in 70% ethanol and removed the genitalia. We also removed a hind leg from each individual. Genital capsules and legs were mounted on slides, and the ovipositor and hind tibia length as a proxy for body size were measured using an Axiocam 208 mounted on a Stemi 508 microscope and accompanying software ZEN 3.5 Blue edition (Zeiss). We report both the absolute and relative (divided by hind tibia length) mean ovipositor length per parasitoid species per gall morphology. To estimate a value representing total mean ovipositor length for each gall morphology, mean length per species was multiplied by abundance per gall morph.

To compare guilds between gall morphologies, we performed a Pearson’s ***χ***^2^-test in R for count data (*chisq.test*) with the variables of mode of parasitism and larval development strategy. We followed each test with calculation of Cramér’s *V*-test of association (√((*χ*^2^/*n*)/min (*k *− 1, *r *− 1)), where *k* is the number of columns (2) and *r* is the number of rows (2), i.e. nominal variables, in the ***χ***^2^ table) [36].

### (c) Parasitoid guild analysis of previously surveyed gall communities

To test the application of these methods in other systems, we repeated the above analysis of guild association with three independent previously published datasets of parasitoid emergence data. As an analogous system to *Aciurina*, we investigated the taxonomically similar enemy communities of the morphologically distinct galls of *Diplolepis rosae* (L.) and *Diplolepis mayri* (Schlechtendal 1877) (Hymenoptera, Diplolepididae) in Hungary and Romania characterized by László & Tóthmérész [15]. Mirroring the *Aciurina* system, these two species are closely related [37] and differ in gall traits that may influence success of different modes of parasitoid attack. The average distance from larval chamber to external gall surface, as represented by gall wall thickness, is significantly greater in galls of *D. mayri* [15]. The second system, characterized by Forbes *et al.* [38], compares the enemy communities of morphologically distinct galls induced by alternating generations of a single host species, *Belonocnema kinseyi* Weld 1921 (formerly synonymized with *Belonocnema treatae* (Mayr 1881)) (Hymenoptera, Cynipidae). This species alternates annually between a sexual generation that induces single-chambered ‘pea’ galls on the leaves, and an asexual generation that induces multi-chambered irregular galls on the roots, of their live oak host plants [39]. The multi-chambered clusters of the sexual gall indicate that the average distance between the inducer larva and the external surface is higher than that in the asexual gall. Guild characterization factors 1 and 2 were determined for parasitoid species by life-history documentation available in the literature for associates of *Diplolepis* [15,40–43] and associates of *Belonocnema* [38,44–50].

To test more broadly in a comparison across multiple gall morphologies and inducer species, we selected a dataset of the 41 129 parasitoid individuals reared from 96 European gall-inducing willow sawfly species of the genus *Euura* characterized by Kopelke *et al*. [51]. This monophyletic genus includes the distinct ‘open gall-makers’, which induce galls between folded or rolled leaf tissue (formerly *Phyllocolpa*), as well as other leaf, petiole, shoot and bud gall-inducing species (including former *Pontania*) [51–53]. This dataset includes guild characterization factors 1 and 2 for all identified parasitoid species to use in our enemy community comparisons. With this multi-species dataset, we aggregated counts per each inducer species + gall morph combination, calculated the proportion of each community assigned to each guild and then compared the mean proportions across gall morph categories (ANOVA).

Finally, to test the corollary to our amended Enemy Hypothesis—enemy communities will have *similar* guild composition (but differ in taxonomic composition) among inducer species with morphologically *similar* galls—we utilize data from *Euura* leaf pea galls. In a study examining seven morphologically similar species of *Euura* leaf pea galls (as *Pontania*), Nyman *et al.* [54] found that, though the gall inducer species were closely related, inducer phylogeny was not a predictor of taxonomic composition of the enemy community, and instead habitat was the strongest factor. With the prior finding that these communities were taxonomically different, but the galls were morphologically similar, we used parasitoid life-history traits compiled by Kopelke *et al.* [51] and compared the guilds from these communities. For all systems, inquiline taxa and parasitoids suspected to be attacking an inquiline host were removed from the datasets. Herein, we report the *χ*^2^, *p*, and Cramér’s *V*-value for *Aciurina*, *Diplolepis, B. kinseyi* and *Euura* datasets.

## 3. Results

### (a) Community composition differences by gall morphology in *Aciurina*

Across all sites and collection years, 24 species were identified as gall associates, including the inducers *A. bigeloviae* (cotton morph) and *A. trixa* (smooth morph) [30]. Overall adult emergence success of *A. bigeloviae* and *A. trixa* was similar (cotton 781, smooth 828). Across both *Aciurina* species, the main observable cause of inducer death was predation by parasitoid wasps; with parasitism 7.78% higher in smooth galls (prop.test, *p* < 0.001). The other main cause of mortality, which was determined by late-season dissections, was desiccation. This was most likely caused by our collection methods, which resulted in the gall no longer remaining connected to the vascular system of the plant. Death due to desiccation occurred in both larval and pupal stages and was higher in cotton galls. Eleven species of wasps, including 10 Chalcidoidea and 1 Ichneumonidae species, are associated as *primary* parasitoids (i.e. parasitoids that utilize *Aciurina* spp. as host) (table 1). The remaining reared associate species and counts of each species are available in [30,55].

**Table 1.** The 11 primary parasitoids of *Aciurina bigeloviae* and *Aciurina trixa* in alphabetical order by family, their modes of parasitism (N = endoparasitoid, C = ectoparasitoid), development strategy (I = idiobiont, K = koinobiont) and host associations displayed as proportion of total individuals reared per host (U = unknown).

species	family	mode of parasitism	development strategy	primary host (% total emergence)
*Baryscapus cecidophagus*	Eulophidae	N	I	*A. trixa* (100%)
*Brasema* sp.	Eupelmidae	C	I	equal (50%)
*Eurytoma bigeloviae*	Eurytomidae	C	I	*A. bigeloviae* (68%)
*Eurytoma chrysothamni*	Eurytomidae	N	K	*A. trixa* (54%)
*Eurytoma contractura*	Eurytomidae	U	U	*A. bigeloviae* (91%)
*Scambus aplopappi*	Ichneumonidae	C	I	*A. bigeloviae* (100%)
*Halticoptera* sp.	Pteromalidae	N	K	*A. trixa* (91%)
*Pteromalus* sp. 1	Pteromalidae	U	U	*A. trixa* (99%)
*Pteromalus* sp. 2	Pteromalidae	U	U	*A. bigeloviae* (76%)
*Torymus capillaceus albitarsis*	Torymidae	C	I	*A. bigeloviae* (67%)
*Torymus citripes*	Torymidae	C	I	*A. bigeloviae* (100%)

**Figure 3.**
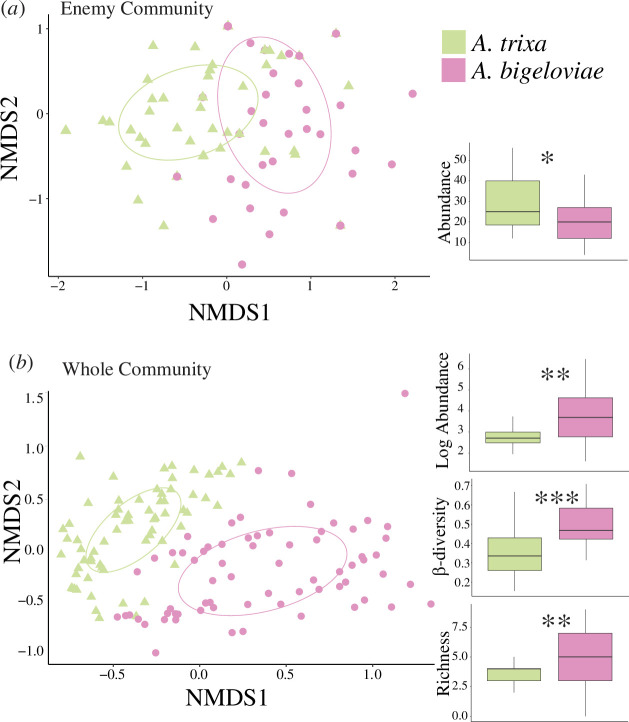
Non-metric multi-dimensional scaling (NMDS) and significant comparisons of β-diversity, abundance and richness between cotton gall *Aciurina bigeloviae* (pink) and smooth gall *Aciurina trixa* (green) gall communities per plant sampled using (*a*) enemy parasitoid community and (*b*) whole arthropod community. For the purpose of visualization, the boxplot for enemy-only communities was generated from counts aggregated by site. Asterisks represent significance of difference (e.g. *p* < 0.05=*, *p* < 0.01=**, *p* < 0.001=***) .

To test the Enemy Hypothesis in the context of this system, we analysed the subset of 11 parasitoid species (hereafter enemy community). Enemy community composition differed between the two gall morphs (PERMANOVA, *p* < 0.01) and there was a good model fit of gall morph on community distances (NMDS, *p* < 0.001, stress = 0.12; figure 3*a*). Even though species overlap is high, there is enough difference in abundances of individual parasitoid species to support that enemy composition can be predicted by gall morph. Smooth gall enemy communities were more abundant (*p* < 0.05), which mirrors the higher rate of parasitism (figure 3*a*). However, richness (*p* = 0.7) and beta-diversity (*p* > 0.05) were not predicted by gall morph.

To further test how gall morphology affects the entire gall-associated community, we performed additional comparisons using all 24 associates. Whole community composition was predicted by gall morph (PERMANOVA, *p* < 0.001), and there was a good NMDS model fit of gall morph on community distances (*p* < 0.001, stress = 0.22; figure 3*b*). Cotton gall communities are more abundant (*p* < 0.01), richer (*p* < 0.01) and more beta-diverse (*p* < 0.001) than smooth gall communities (figure 3*b*). The tomentose nature of *A. bigeloviae* galls may be a major contributor to these differences in community diversity. Leaf structure, especially in relation to pubescence, has been found to have strong effects on general arthropod richness and diversity [56]. Especially in xeric environments, tomentose galls may provide important shelter for plant-dwelling arthropods, further highlighting the potential importance of gall morphology as a driver of microhabitat structure.

### (b) Parasitoid guild characterization in *Aciurina*

From the 11 *Aciurina*-gall enemy species, we were able to classify three parasitoid guilds present in the system based on their mode of parasitism (ectoparasitoid, endoparasitoid), larval development strategy (idiobiont, koinobiont) and ovipositor length (as a proxy for phenology of attack) (table 1). Ectoparasitoids (four species) had a stronger association with cotton galls, and endoparasitoids (three species) had a stronger association with smooth galls (*χ*^2^ = 55.00, *p* < 0.001, Cramér’s *V* = 0.33). Larval development strategy was also correlated to gall type (*χ*^2^ = 23.66, *p* < 0.001, Cramér’s *V* = 0.22). Both variables combined into guilds (as ‘CI’ and ‘NK’, see table 1) were also correlated to gall type (*χ*^2^ = 66.07, *p* < 0.001, Cramér’s *V* = 0.37, figure 5*a*), but this may reflect that all ectoparasitoids were also idiobionts, and two of the three endoparastioids were koinobionts (*Baryscapus cecidophagus* was the only species with an endoparasitic and idiobiotic habit).

**Figure 4.**
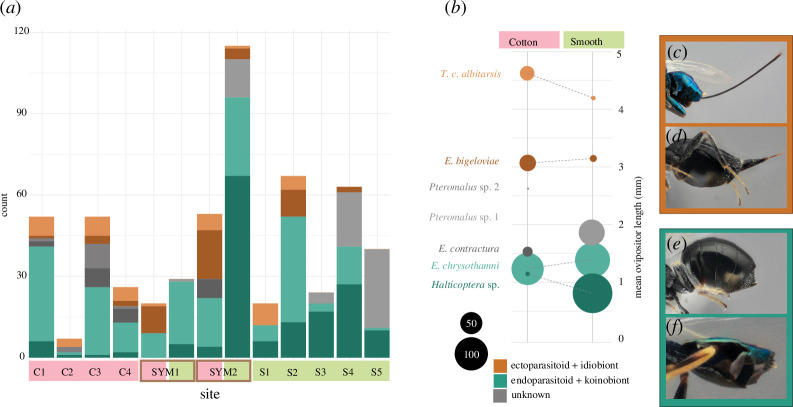
(*a*) Count of emerged individuals of each of the seven most common primary parasitoid species from each site. (*b*) Mean ovipositor length of each of the selected species displayed with points proportional to the number of emerged individuals per gall morphology. (*c*) Lateral view of external ovipositor of *Torymus capillaceus albitarsis*, (*d*) *Eurytoma bigeloviae*, (*e*) *Eurytoma chrysothamni* and (*f*) *Halticoptera* sp. Photo credit Q. Baine.

Because ovipositor length is a continuous variable, we did not assign categories, but endoparasitic + koinobiotic species had short mean lengths (<1.5 mm absolute, <2.0 mm relative) and the second most common of the ectoparasitic + idiobiotic species had long mean lengths (>3.0 mm absolute, >2.5 mm relative) (figure 4*c*–*f*). We found that the average body size of measured parasitoids (as represented by hind tibia length) did not differ as dramatically between gall morphs (mean 0.885 mm smooth versus 0.891 mm cotton). This is consistent with our findings that the hosts *A. trixa* and *A. bigeloviae* have a comparable body size (as represented by wing length; *t.test* female *p* = 0.3, male *p* = 0.26), and therefore provide a similar amount of nutrition to their parasitoids regardless of the difference in gall size [30]. This consistency in parasitoid body size suggests that the ovipositor length of these parasitoids may be an adaptation to gall structures and not just a result of different space or nutritional resources.

### (c) Parasitoid guilds differ between gall morphologies across systems

In the focal species of *Aciurina*, we observed two distinct parasitoid guilds that were each strongly associated with a different gall morphology even in areas of sympatry. The ‘endo + koino + short’ guild (hereafter ‘NKS’) is more prevalent in smooth galls, and ‘ecto + idio + long’ (‘CIL’) guild is more prevalent in cotton galls. Furthermore, we observe a general trend that parasitoid species present in both gall morphologies have shorter ovipositors when associated with smooth galls (figure 4*b*). The total mean ovipositor length of the six most common shared primary parasitoids, weighted by abundance per species, was 1.5 times longer in cotton- than smooth-associated parasitoids (2.11 : 1.44 mm). This pattern may reflect that gall diameter was significantly larger in cotton galls (ANOVA, *p* < 0.001). The differences evident in these two communities support the Enemy Hypothesis.

**Figure 5.**
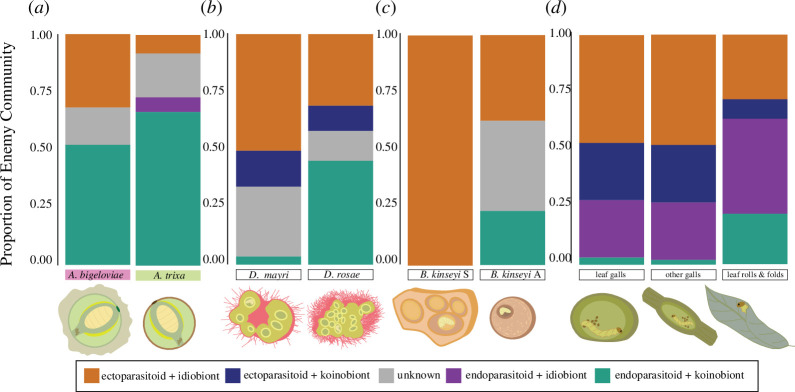
Respective proportions of parasitoid guild per enemy community analysed, with the gall representing shorter larva-to-surface distance on the right side of each graph. Gall representations with larvae are illustrated as cross-sections to show general differences in gall morphology. (*a*) *Aciurina* community [30], (*b*) *Diplolepis* community [15], (*c*) *Belonocnema kinseyi* community [38] and (*d*) *Euura* metacommunity [51].

For the enemies of *D. mayri* and *D. rosae* identified in [15], we characterized the mode of parasitism and larval development strategy for 10 parasitoid species from a total specimen count of 19 601. In this community, all known idiobionts (five species) were also ectoparasitoids (CI); however, the koinobionts (four species) exhibited both modes of parasitism (NK and CK), so at least three well represented guilds were present. Ectoparasitoids (seven species) were more strongly associated with *D. mayri* (*χ*^2^ = 1209, *p* < 0.001, Cramér’s *V* = 0.30) and made up 94% of its parasitoid community compared with 48% of the *D. rosae* community. Larval development strategy was also strongly correlated to gall morph, but with a weaker association (*χ*^2^ = 329.86, *p* < 0.001, Cramér’s *V* = 0.15); the parasitoid community of *D. rosae* was 68% koinobionts (versus 49% for *D. mayri*). Guild was correlated to gall morph as well (*χ*^2^ = 1212.3, *p* < 0.001, Cramér’s *V* = 0.30, figure 5*b*), with the NK guild being the most strongly associated with *D. rosae.*

For the enemy communities of the distinct alternating generations of *Belonocnema kinseyi* galls identified in [38], we saw a similar pattern. Fifteen primary parasitoid species were characterized from a total specimen count of 3596. Of these species, all known endoparasitoids were also koinobionts (NK; three species) and all but one ectoparasitoid were also idiobionts (CI; seven species). The sexual generation root galls were more strongly associated with both ectoparasitism (*χ*^2^ = 506.63, *p* < 0.001, Cramér’s *V* = 0.40) and idiobiosis (*χ*^2^ = 773.35, *p* < 0.001, Cramér’s *V* = 0.50), so were significantly associated with guild CI (*χ*^2^ = 507.49, *p* < 0.001, Cramér’s *V* = 0.41, figure 5*c*). Though other factors may be at play in comparing alternate generations (e.g. seasonality), the pattern that different galls host different enemy communities, even when the inducers are conspecific, indicates that gall morphology may be a greater influence on guild assembly than inducer phylogeny.

Of the 102 identified parasitoid species of galling *Euura*, all four guild combinations were represented (CI: 46, CK: 12, NI: 4 and NK: 40). We merged gall morphology type categories that overlapped or were reasonably grouped from a standpoint of potentially defensive external characters (e.g. leaf ‘pea’ gall, leaf ‘sausage’ gall, leaf ‘bean’ gall) and ended up with five morphological gall categories: bud, petiole, shoot, leaf and leaf fold/roll. The mean proportion per inducer community of both mode of parasitism and larval development strategy significantly differed among the five categories (*F* = 115.8, *p* < 0.0001; *F* = 4.513, *p* < 0.001, respectively). *Post hoc* comparison (*TukeyHSD*) indicated that leaf fold/roll galls hosted a significantly higher proportion of endoparasitoids than all other categories, and a higher proportion of koinobionts than either the leaf or bud category. In examining total counts among the five categories, we found significant difference and strong association of mode of parasitism (*χ*^2^ = 11646.3, *p* < 0.0001, Cramér’s *V* = 0.60), and significant difference but weak association of larval strategy (*χ*^2^ = 292.95, *p* < 0.0001, Cramér’s *V* = 0.09); however, the most significant difference and strong association was found in guild (*χ*^2^ = 25246.68, *p* < 0.0001, Cramér’s *V* = 0.78, figure 5*d*). Interestingly, the most significant difference observable in guild assembly across a sampling of 96 species of this clade is between the communities associated with leaf fold and leaf roll galls, and those associated with all others. In the evolution of European *Euura*, gall form generally follows phylogeny [57], and so, unlike our other systems, these results support closely related species having more similar associate enemies. However, we observe a familiar pattern: the leaf fold and leaf roll galls have both a much shorter larva-to-surface distance (as short as the thickness of a leaf, and often open on one end [52,58]) and relatively fewer ectoparasitoids and idiobionts than their more protected relatives in galls with thicker walls and internal air space.

In the study of the morphologically similar *Euura* pea leaf galls by Nyman *et al.* [54], we found that, of the primary parasitoid species that could be characterized to guild (97.7% of total individuals), the CI guild was extremely dominant (99.7%) in the aggregate community. Difference in guild among the seven inducers was not significant and association was low (*χ*^2^ = 10.49, *p* = 0.1, Cramér’s *V* = 0.13). Only one inducer of the seven was host to a parasitoid species characterized in the guild NK, *Euura aquilonis* (Benson, 1941), and that parasitoid only made up 2% of its total enemies. This result of almost completely uniform guild composition indicates that highly similar gall morphologies have highly similar enemy guilds across habitats, even where the taxonomic identity of enemies has changed.

## 4. Discussion

Our results provide robust support for the modification of the Enemy Hypothesis by demonstrating that gall morphology significantly influences associated parasitoid functional guild composition. This pattern suggests that gall traits function as defensive adaptations against particular guilds of natural enemies, which can indirectly affect the taxonomic composition and attack rate of parasitoids. Furthermore, the methodology outlined here to test the modified hypothesis is applicable to a wide range of galling systems and can be employed globally. We hope this study provides a compelling case for the importance of considering enemy functional guilds in the examination of potentially defensive traits.

The Enemy Hypothesis considers the gall in the context of its rich community of predators, inquilines and parasitoids, many of which exhibit specialized adaptations that allow successful gall attack, and states that variation in external gall structure is explained by selection against predatory organisms that target the immature gall inducer [9,59]. Support for this hypothesis is strong and is best exemplified in galling taxa with high structural diversity, e.g. cynipoid wasps [9,10,17,24,25]. Previous studies have tested the central prediction that different gall morphologies will have (i) distinctions in the taxonomy of gall-associated enemy communities and (ii) variations in the rate of parasitism [19,22]; however, this can be improved by considering how galls influence ecological interactions between the inducer and parasitoids. In this study, we amend the central prediction to incorporate this crucial aspect by adding a third component—that distinct gall morphs will have differences in enemy guild assembly (figure 1).

**Figure 6.**
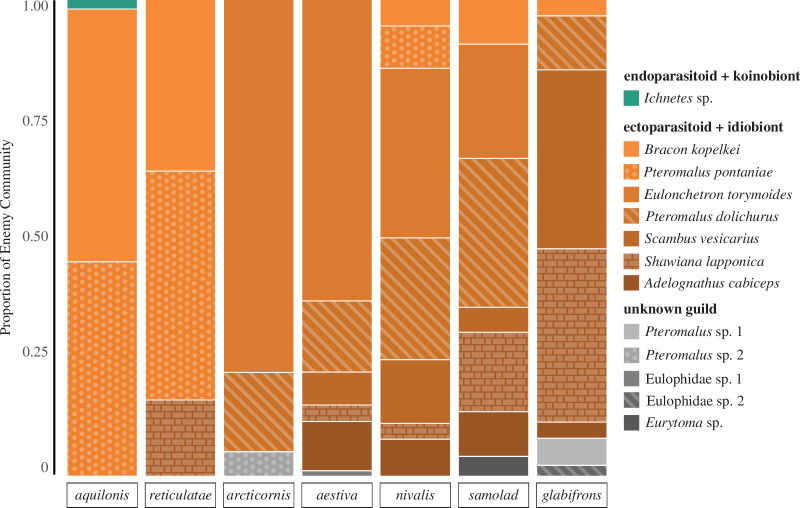
Respective proportions of parasitoid species and guild per enemy community of each of seven morphologically similar *Euura* (as *Pontania*) inducer species leaf pea galls, adapted from Nyman *et al.* [54].

We found strong support for this added component by demonstrating that gall morphology influences parasitoid functional guild composition. Specifically, there are significant differences in enemy guild composition in closely related gall inducers that generate distinct external gall morphologies (figure 5), whereas morphologically similar galls have shared enemy guild compositions (figure 6). Across four independent gall systems analysed for parasitoid guild composition, we observed a consistent pattern (figure 5): galls with a longer average larva-to-surface distance (*A. bigeloviae*, *D. mayri,* the sexual generation of *B. kinseyi*, and closed galls of *Euura* sawflies) had significantly more CI guild parasitoids, whereas galls with a shorter average larva-to-surface distance (*A. trixa*, *D. rosae,* asexual *B. kinseyi*, and leaf fold + roll galls of *Euura* sawflies) had significantly more NK guild parasitoids. Previous research from several diverse gall systems supports that larva-to-surface distance is influential in gall parasitism susceptibility (e.g. oak and chestnut cynipid wasps, willow sawflies, *Eurosta* and *Urophora* tephritid flies [60–64]); however, our findings indicate that larva-to-surface distance is additionally a critical factor in shaping enemy *guild* composition.

While it is enticing to generate hypotheses linking certain gall traits to particular enemy guilds, it remains unclear which traits or combinations of traits may select for different parasitoid guild success. For example, among the taxa examined here the larva-to-surface distance is maintained by different traits: the *A. bigeloviae* gall has dense, cotton-like tomentum, whereas the *D. mayri* gall has woody parenchymatous tissue [15]. Furthermore, there are numerous traits unconnected to larva-to-surface distance, such as the sticky and/or waxy resin of *A. trixa* galls, which also likely influence how parasitoids interact with the gall. This highlights not only the diversity of effects that top-down evolutionary pressure can have on the extended phenotype of a host but also the complex defensive landscape that gall-associated enemies navigate.

Accurately linking certain gall traits to particular enemy guilds will require systematic characterization of gall traits such as hairiness, induction delay, wall thickness and chemical exudate composition (e.g. [10]). A necessary next step, we believe, in testing this hypothesis would be to identify the enemy communities in taxonomically distant gall inducers with convergent gall morphologies. For example, *A. bigeloviae* (Diptera: Tephritidae) and *Asphondylia neomexicana* (Cockerell 1896) (Diptera: Cecidomyiidae) are members of two independent origins of gall induction within Diptera and utilize distantly related host plants (*E. nauseosa* and *Atriplex canescens* (Pursh) Nutt., respectively); however, they have almost identical cottony galls that have overlapping ranges in New Mexico. Because of these shared characteristics, we would predict that *A. bigeloviae*’s enemy guild composition would closely resemble that of the very distantly related gall midge *A. neomexicana*.

However, testing hypotheses in cecidology related to enemy guild presents several additional challenges. First, few existing community surveys are able to provide any life-history data on parasitoid taxa. Second, non-specific identification of enemies (e.g. ‘*Aprostocetus* sp.’)—particularly in the highly diverse and gall-associated Chalcidoidea families Eulophidae, Eurytomidae and Pteromalidae—does not reliably predict life-history characteristics [46,65]. This means our augmented ‘Enemy *Guild* Hypothesis’ cannot be applied to many previously published studies on gall enemy communities [e.g. 66–72]; however, with the methodology outlined in this work, we hope that data collection moving forward in gall-associated communities can incorporate these crucial elements.

The pattern we observed in enemy guild composition was robust even between *A. trixa* and *A. bigeloviae*, which are sister taxa, are geographically sympatric, and have phenological overlap. Strikingly, among enemy species present on both gall morphologies, the ovipositor length was on average 1.5 times longer on larger *A. bigeloviae* (cotton) galls, without a corresponding increase in overall body size. Though we did not directly test for the causes of this difference in size, possibilities include enemy trait plasticity or ecological fitting that allows for preferred host selection, or that these enemies may be *en route* to specialization to different gall morphs as a consequence of cascading speciation (i.e. strict co-dispersal, host tracking) [73,74].

These insights not only enhance our understanding of the evolutionary dynamics between galling insects and their parasitoids but also highlight the broader ecological implications of symbiotic interactions as drivers of biodiversity and ecosystem complexity. A gall structure is a de novo habitat, similar to a coral reef, that hosts a unique food web not mirrored elsewhere in the greater ecosystem [75] and generally hosts a richer and more abundant suite of associates than other herbivorous insects [11,16]. External gall traits are highly diverse across galling species and include striking adaptations (e.g. spines, nectar secretion, detachable inner chambers); in this work, we have described general trends that may potentially help to better understand the evolution of these complex structures and predict enemy community biodiversity. Evidence that supports the Enemy Hypothesis on a functional guild level further cements the assertion that gall-inducing insects are anchors of biodiversity; the ongoing emergence of novel gall morphologies generates fresh niches for parasitoid taxa to adapt to and exploit. By measuring how gall traits affect associated arthropod community structure, the field might begin to better estimate how these underexplored symbiosis-engineered ecosystems affect biodiversity.

## Data Availability

Species count data and R code used to generate results and base plots included in this manuscript are publicly available on Figshare [76].

## References

[1] Borges RM. 2017 Co-niche construction between hosts and symbionts: ideas and evidence. J. Genet. **96**, 483–489. (10.1007/s12041-017-0792-9) 28761011

[2] Chomicki G, Weber M, Antonelli A, Bascompte J, Kiers ET. 2019 The impact of mutualisms on species richness. Trends Ecol. Evol. **34**, 698–711. (10.1016/j.tree.2019.03.003) 31003875

[3] Chomicki G, Beinart R, Prada C, Ritchie KB, Weber MG. 2022 Editorial: symbiotic relationships as shapers of biodiversity. Front. Ecol. Evol. **10**, 850572. (10.3389/fevo.2022.850572)

[4] Cornwallis CK, van ’t Padje A, Ellers J, Klein M, Jackson R, Kiers ET, West SA, Henry LM. 2023 Symbioses shape feeding niches and diversification across insects. Nat. Ecol. Evol. **7**, 1022–1044. (10.1038/s41559-023-02058-0) 37202501 PMC10333129

[5] Kurtz J, Schulenburg H, Reusch TBH. 2016 Host–parasite coevolution-rapid reciprocal adaptation and its genetic basis. Zoology **119**, 241–243. (10.1016/j.zool.2016.06.011) 27432487

[6] Ponge J. 2021 Communities, ecosystem engineers, and functional domains. Ecol. Res. **36**, 766–777. (10.1111/1440-1703.12247)

[7] Abrahamson WG, Sattler JF, McCrea KD, Weis AE. 1989 Variation in selection pressures on the goldenrod gall fly and the competitive interactions of its natural enemies. Oecologia **79**, 15–22. (10.1007/BF00378234) 28312807

[8] Luz FA. 2019 Guilds in insect galls: who is who. FL Entomol. **102**, 207. (10.1653/024.102.0133)

[9] Stone GN, Schönrogge K. 2003 The adaptive significance of insect gall morphology. Trends Ecol. Evol. **18**, 512–522. (10.1016/S0169-5347(03)00247-7)

[10] Bailey R, Schönrogge K, Cook JM, Melika G, Csóka G, Thuróczy C, Stone GN. 2009 Host niches and defensive extended phenotypes structure parasitoid wasp communities. PLoS Biol. **7**, e1000179. (10.1371/journal.pbio.1000179) 19707266 PMC2719808

[11] Cornelissen T, Cintra F, Santos JC. 2016 Shelter-building insects and their role as ecosystem engineers. Neotrop. Entomol. **45**, 1–12. (10.1007/s13744-015-0348-8) 26631227

[12] Whitham TG *et al*. 2003 Community and ecosystem genetics: a consequence of the extended phenotype. Ecology **84**, 559–573. (10.1890/0012-9658(2003)084[0559:CAEGAC]2.0.CO;2)

[13] Martinson EO, Werren JH, Egan SP. 2022 Tissue-specific gene expression shows a cynipid wasp repurposes oak host gene networks to create a complex and novel parasite-specific organ. Mol. Ecol. **31**, 3228–3240. (10.1111/mec.16159) 34510608

[14] Cornell HV. 1983 The secondary chemistry and complex morphology of galls formed by the Cynipinae (Hymenoptera): why and how? Am. Midl. Nat. **110**, 225–234. (10.2307/2425263)

[15] László Z, Tóthmérész B. 2013 The enemy hypothesis: correlates of gall morphology with parasitoid attack rates in two closely related rose cynipid galls. Bull. Entomol. Res. **103**, 326–335. (10.1017/S0007485312000764) 23217451

[16] Price PW, Fernandes GW, Waring GL. 1987 Adaptive nature of insect galls. Environ. Entomol. **16**, 15–24. (10.1093/ee/16.1.15)

[17] László Z, Sólyom K, Prázsmári H, Barta Z, Tóthmérész B. 2014 Predation on rose galls: parasitoids and predators determine gall size through directional selection. PLoS One **9**, e99806. (10.1371/journal.pone.0099806) 24918448 PMC4053394

[18] Ito M, Hijii N. 2004 Roles of gall morphology in determining potential fecundity and avoidance of parasitoid attack in Aphelonyx glanduliferae . J. For. Res. **9**, 93–100. (10.1007/s10310-003-0057-8)

[19] Price PW, Pschorn‐Walcher H. 1988 Are galling insects better protected against parasitoids than exposed feeders?: A test using tenthredinid sawflies. Ecol. Entomol. **13**, 195–205. (10.1111/j.1365-2311.1988.tb00347.x)

[20] Schönrogge K, Stone GN, Crawley MJ. 1996 Alien herbivores and native parasitoids: rapid developments and structure of the parasitoid and inquiline complex in an invading gall wasp Andricus quercuscalicis (Hymenoptera: Cynipidae) . Ecol. Entomol. **21**, 71–80. (10.1111/j.1365-2311.1996.tb00268.x)

[21] Luz FA, Goetz APM, Mendonça Jr M de S. 2020 Phenotypic matching in ovipositor size in the parasitoid Galeopsomyia sp. (Hymenoptera, Eulophidae) attacking different gall inducers. Iheringia Sér. Zool. **110**, e2020008. (10.1590/1678-4766e2020008)

[22] Luz FA, Goetz APM, Mendonça M de S Jr. 2021 What drives gallers and parasitoids interacting on a host plant? A network approach revealing morphological coupling as the main factor. Ecol. Entomol. **46**, 334–341. (10.1111/een.12967)

[23] Dixon KA, Lerma RR, Craig TP, Hughes KA. 1998 Gall morphology and community composition in Asphondylia flocossa (Cecidomyiidae) galls on Atriplex polycarpa (Chenopodiaceae). Environ. Entomol. **27**, 592–599. (10.1093/ee/27.3.592)

[24] Schönrogge K, Stone GN, Crawley MJ. 1995 Spatial and temporal variation in guild structure: parasitoids and inquilines of Andricus quercuscalicis (Hymenoptera: Cynipidae) in its native and alien ranges. Oikos **72**, 51–60. (10.2307/3546037)

[25] Ward AKG *et al*. 2022 The arthropod associates of 155 North American cynipid oak galls. Zool. Stud. **61**, e57. (10.6620/ZS.2022.61-57) 36644628 PMC9810845

[26] Mills NJ. 1994 Parasitoid guilds: defining the structure of the parasitoid communities of endopterygote insect hosts. Environ. Entomol. **23**, 1066–1083. (10.1093/ee/23.5.1066)

[27] Hawkins BA, Mills NJ. 1996 Variability in parasitoid community structure. J. Anim. Ecol. **65**, 501. (10.2307/5785)

[28] Mayhew PJ, Blackburn TM. 1999 Does development mode organize life‐history traits in the parasitoid Hymenoptera? J. Anim. Ecol. **68**, 906–916. (10.1046/j.1365-2656.1999.00338.x)

[29] Blondel J. 2003 Guilds or functional groups: does it matter? Oikos **100**, 223–231. (10.1034/j.1600-0706.2003.12152.x)

[30] Baine Q, Casares EE, Hughes DWW, Martinson VG, Martinson EO. 2024 Arthropod communities associated with gall-inducing Aciurina bigeloviae and Aciurina trixa (Diptera: Tephritidae) in New Mexico . Ann. Entomol. Soc. Am. **117**, 107–117. (10.1093/aesa/saad037)

[31] Clarke KR, Gorley RN. 2015 PRIMER v7: user manual/tutorialPlymouth, UK: PRIMER-E. See https://learninghub.primer-e.com/books/primer-v7-user-manual-tutorial.

[32] Anderson MJ, Gorley RN, Clarke KR. 2008 PERMANOVA+ for PRIMER: guide to software and statistical methodsPlymouth, UK: PRIMER-E. See https://learninghub.primer-e.com/books/permanova-for-primer-guide-to-software-and-statistical-methods.

[33] R Core Team. 2021 *R: a language and environment for statistical computing*. See http://www.R-project.org/.

[34] Oksanen J *et al*. 2016 Vegan: community ecology package. See https://cran.r-project.org/web/packages/vegan/index.html.

[35] Bates D, Mächler M, Bolker B, Walker S. 2015 Fitting linear mixed-effects models using lme4. J. Stat. Softw. **67**, 1–48. (10.18637/jss.v067.i01)

[36] Acock AC, Stavig GR. 1979 A measure of association for nonparametric statistics. Social Forces **57**, 1381–1386. (10.2307/2577276)

[37] Zhang YM, Buffington ML, Looney C, László Z, Shorthouse JD, Ide T, Lucky A. 2020 UCE data reveal multiple origins of rose gallers in North America: global phylogeny of Diplolepis geoffroy (Hymenoptera: Cynipidae). Mol. Phylogenet. Evol. **153**, 106949. (10.1016/j.ympev.2020.106949) 32866614

[38] Forbes AA, Hall MC, Lund J, Hood GR, Izen R, Egan SP, Ott JR. 2016 Parasitoids, hyperparasitoids, and inquilines associated with the sexual and asexual generations of the gall former, Belonocnema treatae (Hymenoptera: Cynipidae). Ann. Entomol. Soc. Am. **109**, 49–63. (10.1093/aesa/sav112)

[39] Zhang YM, Egan SP, Driscoe AL, Ott JR. 2021 One hundred and sixty years of taxonomic confusion resolved: Belonocnema (Hymenoptera: Cynipidae: Cynipini) gall wasps associated with live oaks in the USA . Zool. J. Linn. Soc. **193**, 1234–1255. (10.1093/zoolinnean/zlab001)

[40] Rizzo MC, Massa B. 2006 Parasitism and sex ratio of the Bedeguar gall wasp Diplolepis rosae (L.) (Hymenoptera: Cynipidae) in Sicily (Italy). J. Hymenopt. Res. **15**, 277–285.

[41] Gómez JF, Nieves-Aldrey JL, Nieves MH, Stone GN. 2011 Comparative morphology and biology of terminal instar larvae of some Eurytoma (Hymenoptera, Eurytomidae) species parasitoids of gall wasps (Hymenoptera, Cynipidae) in Western Europe. Zoosystema **33**, 287–323. (10.5252/z2011n3a3)

[42] László Z, Tóthmérész B. 2011 Parasitism, phenology and sex ratio in galls of Diplolepis rosae in the eastern Carpathian Basin. Entomol. Rom. **16**, 33–38.

[43] Gómez JF, Nieves-Aldrey JL. 2012 Notes on the larval morphology of Pteromalidae (Hymenoptera: Chalcidoidea) species parasitoids of gall wasps (Hymenoptera: Cynipidae) in Europe. Zootaxa **3189**, 39–55. (10.11646/zootaxa.3189.1.3)

[44] Askew RR. 1961 Some biological notes on the pteromalid (Hym., Chalcidoidea) genera Caenacis Förter, Cecidostiba Thomson and Hobbya Delucchi, with descriptions of two new species. Entomophaga **6**, 57–67. (10.1007/BF02373205)

[45] Baur H, Kranz-Baltensperger Y, Cruaud A, Rasplus JY, Timokhov AV, Gokhman VE. 2014 Morphometric analysis and taxonomic revision of Anisopteromalus Ruschka (Hymenoptera: Chalcidoidea: Pteromalidae) - an integrative approach. Syst. Entomol. **39**, 691–709. (10.1111/syen.12081) 26074661 PMC4459240

[46] Gibson GAP, Huber JT, Woolley JB (eds). 1997 Annotated keys to the genera of nearctic Chalcidoidea (Hymenoptera). Ottawa, Canada: NRC Research Press.

[47] Gil‐Tapetado D, Durán‐Montes P, García‐París M, López‐Estrada EK, Sánchez‐Vialas A, Jiménez‐Ruiz Y, Gómez JF, Nieves‐Aldrey JL. 2022 Host specialization is ancestral in Torymus (Hymenoptera, Chalcidoidea) cynipid gall parasitoids . Zool. Scr. **51**, 91–118. (10.1111/zsc.12515)

[48] Gómez JF, Nieves-Aldrey JL, Stone GN. 2013 On the morphology of the terminal-instar larvae of some European species of Sycophila (Hymenoptera: Eurytomidae) parasitoids of gall wasps (Hymenoptera: Cynipidae). J. Nat. Hist. **47**, 2937–2960. (10.1080/00222933.2013.791937)

[49] de Macêdo MV, Monteiro RF. 1989 Seed predation by a braconid wasp, Allorhogas sp. (Hymenoptera). J. NY Entomol. Soc. **97**, 358–362.

[50] Sheikh SI, Ward AKG, Zhang YM, Davis CK, Zhang L, Egan SP, Forbes AA. 2022 Ormyrus labotus (Hymenoptera: Ormyridae): another generalist that should not be a generalist is not a generalist. Insect Syst. Divers. **6**, 8. (10.1093/isd/ixac001)

[51] Kopelke JP, Nyman T, Cazelles K, Gravel D, Vissault S, Roslin T. 2017 Food-web structure of willow-galling sawflies and their natural enemies across Europe. Ecology **98**, 1730. (10.1002/ecy.1832) 28369917

[52] Liston AD, Heibo E, Prous M, Vårdal H, Nyman T, Vikberg V. 2017 North European gall-inducing Euura sawflies (Hymenoptera, Tenthredinidae, Nematinae). Zootaxa **4302**, 1–115. (10.11646/zootaxa.4302.1.1)

[53] Nyman T. 2000 Phylogeny and ecological evolution of gall-inducing sawflies (Hymenoptera: Tenthredinidae). PhD Dissertation, University of Joensuu, Finland.

[54] Nyman T, Leppänen SA, Várkonyi G, Shaw MR, Koivisto R, Barstad TE, Vikberg V, Roininen H. 2015 Determinants of parasitoid communities of willow-galling sawflies: habitat overrides physiology, host plant and space. Mol. Ecol. **24**, 5059–5074. (10.1111/mec.13369) 26340615

[55] Baine Q, Casares EE, Carabotta E, Martinson VG, Martinson EO. 2023 Galls on galls: a hypergall-inducing midge and its parasitoid community. Ecology **104**, e4018. (10.1002/ecy.4018) 36883213

[56] Walter DE. 1996 Living on leaves: mites, tomenta, and leaf domatia. Annu. Rev. Entomol. **41**, 101–114. (10.1146/annurev.en.41.010196.000533) 15012326

[57] Nyman T, Roininen H, Vuorinen JA. 1998 Evolution of different gall types in willow-feeding sawflies (Hymenoptera: Tenthredinidae). Evolution **52**, 465–474. (10.1111/j.1558-5646.1998.tb01646.x) 28568332

[58] Smith DR, Fritz RS. 1996 Review of the eastern United States species of the leaf-folding sawflies of the genus Phyllocolpa Benson (Hymenoptera: Tenthredinidae). Proc. Entomol. Soc. Wash. **98**, 695–707.

[59] Bouletreau M. 1986 The genetic and coevolutionary interactions between parastoids and their hosts. In Insect parasitoids: 13th Symposium of the Royal Entomological Society of London, 18–19 September 1985, Imperial College, London (eds J Waage, DJ Greathead), pp. 225–264. London, UK: Academic Press.

[60] Cooper WR, Rieske LK. 2010 Gall structure affects ecological associations of Dryocosmus kuriphilus (Hymenoptera: Cynipidae). Environ. Entomol. **39**, 787–797. (10.1603/EN09382) 20550791

[61] Ito M, Hijii N. 2004 Relationships among abundance of galls, survivorship, and mortality factors in a cynipid wasp, Andricus moriokae (Hymenoptera: Cynipidae). J. For. Res. **9**, 355–359. (10.1007/s10310-004-0091-1)

[62] Price PW, Clancy KM. 1986 Interactions among three trophic levels: gall size and parasitoid attack. Ecology **67**, 1593–1600. (10.2307/1939090)

[63] Weis AE, Abrahamson WG. 1985 Potential selective pressures by parasitoids on a plant‐herbivore interaction. Ecology **66**, 1261–1269. (10.2307/1939179)

[64] Zwölfer H, Böheim M, Beck E. 2015 Eurytoma serratulae and E. robusta (Hymenoptera, Eurytomidae): complementary host exploitation strategies of coexisting parasitoids and their impact on the host Urophora cardui. J. Hymenopt. Res. **42**, 47–62. (10.3897/JHR.42.8847)

[65] Burks R *et al*. 2022 From hell’s heart I stab at thee! A determined approach towards a monophyletic Pteromalidae and reclassification of Chalcidoidea (Hymenoptera). J. Hymenopt. Res. **94**, 13–88. (10.3897/jhr.94.94263)

[66] Eliason EA, Potter DA. 2000 Biology of Callirhytis cornigera (Hymenoptera: Cynipidae) and the arthropod community inhabiting its galls. Environ. Entomol. **29**, 551–559. (10.1603/0046-225X-29.3.551)

[67] Güçlü S, Hayat R, Shorthouse JD, Tozlu G. 2008 Gall-inducing wasps of the genus Diplolepis (Hymenoptera: Cynipidae) on shrub roses of Turkey. Proc. Entomol. Soc. Wash. **110**, 204–217. (10.4289/0013-8797-110.1.204)

[68] López-Núñez FA, Ribeiro S, Marchante H, Heleno RH, Marchante E. 2019 Life inside a gall: diversity, phenology and structure of Portuguese gall communities, their hosts, parasitoids and inquilines. Arthropod Plant Interact. **13**, 477–488. (10.1007/s11829-018-9655-4)

[69] Mete Ö, Mergen YO. 2017 The community components associated with two common rose gall wasps (Hymenoptera: Cynipidae: Diplolepidini) in Turkey. Turk. J. Zool. **41**, 696–701. (10.3906/zoo-1602-20)

[70] Serrano-Muñoz M, Pujade-Villar J, Lobato-Vila I, Valencia-Cuevas L, Mussali-Galante P, Castillo-Mendoza E, Callejas-Chavero A, Tovar-Sánchez E. 2022 Influence of elevation gradient on cynipid galls and their associated insect communities: the case of Quercus rugosa (Fagaceae). Arthropod Plant Interact. **16**, 401–421. (10.1007/s11829-022-09911-2)

[71] Stone GN, Schönrogge K, Crawley MJ, Fraser S. 1995 Geographic and between-generation variation in the parasitoid communities associated with an invading gallwasp, Andricus quercuscalicis (Hymenoptera: Cynipidae). Oecologia **104**, 207–217. (10.1007/BF00328585) 28307357

[72] Weinersmith KL, Forbes AA, Ward AKG, Brandão-Dias PFP, Zhang YM, Egan SP. 2020 Arthropod community associated with the asexual generation of Bassettia pallida (Hymenoptera: Cynipidae). Ann. Entomol. Soc. Am. **113**, 373–388. (10.1093/aesa/saaa009)

[73] Bunnefeld L, Hearn J, Stone GN, Lohse K. 2018 Whole-genome data reveal the complex history of a diverse ecological community. Proc. Natl Acad. Sci. USA **115**, E6507–E6515. (10.1073/pnas.1800334115) 29946026 PMC6048486

[74] Stireman JO, Nason JD, Heard SB, Seehawer JM. 2006 Cascading host-associated genetic differentiation in parasitoids of phytophagous insects. Proc. R. Soc. B **273**, 523–530. (10.1098/rspb.2005.3363) PMC1560066 16537122

[75] Sanders D, Jones CG, Thébault E, Bouma TJ, van der Heide T, van Belzen J, Barot S. 2014 Integrating ecosystem engineering and food webs. Oikos **123**, 513–524. (10.1111/j.1600-0706.2013.01011.x)

[76] Baine Q, Martinson EO, Martinson VG. 2024 Data from: Enemies of galls. Figshare. (10.6084/m9.figshare.25374379.v1)

